# Age-based spatial distribution of workers is resilient to worker loss in a subterranean termite

**DOI:** 10.1038/s41598-022-11512-1

**Published:** 2022-05-12

**Authors:** Sang-Bin Lee, Thomas Chouvenc, Nobuaki Mizumoto, Aaron Mullins, Nan-Yao Su

**Affiliations:** 1 Department of Entomology and Nematology, Ft. Lauderdale Research and Education Center, University of Florida, 3205 College Avenue, Ft. Lauderdale, FL 33314 USA; 2 Okinawa Institute of Science and Technology Graduate University, Onna-son, Okinawa 940-0495 Japan

**Keywords:** Animal behaviour, Behavioural ecology, Social evolution

## Abstract

Elaborate task allocation is key to the ecological success of eusocial insects. Termite colonies are known for exhibiting age polyethism, with older instars more likely to depart the reproductive center to access food. However, it remains unknown how termites retain this spatial structure against external disturbances. Here we show that a subterranean termite *Coptotermes formosanus* Shiraki combines age polyethism and behavioral flexibility to maintain a constant worker proportion at the food area. Since this termite inhabits multiple wood pieces by connecting them through underground tunnels, disastrous colony splitting events can result in the loss of colony members. We simulated this via weekly removal of all individuals at the food area. Our results showed that termites maintained a worker proportion of ~ 20% at the food area regardless of changes in total colony size and demographic composition, where younger workers replaced food acquisition functions to maintain a constant worker proportion at the food area. Food consumption analysis revealed that the per-capita food consumption rate decreased with younger workers, but the colony did not compensate for the deficiency by increasing the proportion of workers at the feeding site. These results suggest that termite colonies prioritize risk management of colony fragmentation while maintaining suitable food acquisition efficiency with the next available workers in the colony, highlighting the importance of task allocation for colony resiliency under fluctuating environments.

## Introduction

In eusocial insects, task allocation operates through the collective behavior of individuals without any hierarchical control^1,2^. Task allocation within a colony can be maintained by a combination of mechanisms, including response threshold variance^3–6^, spatial distribution^7,8^, and age polyethism^9,10^. This decentralized task allocation allows the colony to perform multiple tasks simultaneously with individuals temporarily dedicated to particular tasks. Models of decentralized distribution systems suggest that the task allocation mechanisms in social insects could achieve resiliency^11,12^. In other words*,* colonies can maintain their function even if some individuals fail to perform tasks or are simply missing^13–16^.

The maintenance of food acquisition functions in the colony is essential to provide resources to the colony. But at the same time, food acquisition can be a risky task. In social Hymenoptera, foragers have to leave the safety of the nest to look for food resources and are exposed to risks outside the colony such as pathogens, competitors, and predators^17,18^. Thus, the loss of foragers is inevitable. Colonies alleviate such impact through task re-allocation. For example, workers can alter their behaviors in response to changes in external or internal conditions of the colony^13,19–22^. Indeed, manipulative studies from eusocial Hymenoptera demonstrated that when individuals involved in particular tasks were removed from a colony, they were subsequently replaced by some of the remaining individuals^23–25^. This replacement of removed workers by other workers revealed that behavioral flexibility at the individual level could maintain effective task performance at the colony level^12^. Hence, task allocation mechanisms in insect societies ultimately allow for colony resiliency against disturbances^26^.

Termites have evolved eusociality through a different evolutionary pathway from social Hymenoptera^27^, but they also display age polyethism and task allocation^28–38^. Subterranean termites, or multiple-site nesting termites, nest over multiple pieces of wood resources by interconnecting them through underground tunnels^39–43^. Among physically isolated multiple nests, only one could contain the primary reproductives, while others may or may not house supplementary reproductives in addition to the remaining castes, mostly workers which are found throughout^40^. In the colony, younger workers tend to remain close to reproductives, while older workers are distributed farther away from reproductives^30,44^. Thus, the spatial distribution of colony members can be interpreted as a task allocation because workers with reproductives may focus on brood care work, while workers apart from reproductives may carry out food acquisition. Despite thousands of workers present in the colony, only a small portion (e.g., 10 to 20%) of them remain apart from the area with reproductives at any given time^44,45^. How do termite colonies achieve this spatial organization? This could simply reflect the age composition of the colony, indicating the colony has 10 to 20% old workers which depart from the reproductive area. Or this might result from active regulation of individual behavior, indicated by workers of mixed age leaving the reproductive area.

To test this idea, we focused on the loss of colony members in subterranean termites. For subterranean termites, there are risks to departing the area where reproductives are present. The tunnel path to the separate wood pieces can be disconnected by natural disturbances such as flooding events^46^ or exposed to higher loss to predation^47^. Therefore, subterranean termite colonies can recurrently experience an unpredictable loss of older colony members that are at feeding sites. Simulating such loss events can provide the opportunity not only to characterize the task allocation processes in termites, but also to understand how a termite colony could maintain colony function against disturbances.

In this study, we used *Coptotermes formosanus*, which is a subterranean termite and one of the most widely studied termite species due to economic importance as this termite not only causes damage to wooden structures, but also has invaded many different regions worldwide^48,49^. We address three different questions: if all termites are removed from the food area, (1) would remaining workers come out of the feeding site to access food?, (2) how would it alter the proportion and composition of workers at the feeding site?, and (3) how would it impact the food intake of the colony?. We first hypothesized that the loss of workers at the food area would result in their replacement by the next age cohort of workers. As younger workers may not be as efficient as older workers in food provisioning, we then investigated if colonies would increase the proportion of workers at feeding sites to compensate for the loss of workers to maintain colony functions.

## Results

### Changes in worker demographics at the food area after removal events

As termites at the food area were removed repeatedly, the total number of termites in the colony continue to decline over time (Table 1). As a result, colony size decreased by almost half of the initial size by the end of the experiment after the four consecutive removal events (worker loss of 46.39 ± 0.46%). A few hours after removals, workers and soldiers moved from the reproductive area through the re-connected tubing to the food area. Even after repeated removals and loss of colony members, termites always resumed activity at the food area when provided with access to a new wood source.

**Table 1:** Average total number of workers (Mean ± SEM) in the colony, average number of workers at the food area, and average worker proportion at the food area during the experiment in the *Coptotermes formosanus* colony (*n* = 4 colonies, 3yrs-old).

	1st removal	2nd removal	3rd removal	4th removal	Statistics	*P*
Average total number of workers in the colony at the time of the removal	6297.75 ± 487.74	5009.00 ± 422.95	4078.00 ± 348.6	3291.75 ± 309.32		
Average number of workers at the food area (removed)	1288.75 ± 155.32	931.00 ± 91.13	786.25 ± 49.16	670.50 ± 63.64	χ^2^: 8.58	0.035
Average worker proportions at the food area	0.20 ± 0.02	0.19 ± 0.01	0.20 ± 0.01	0.20 ± 0.01	*F*: 0.0223	0.88

Average worker proportions at the food area were determined using the number of workers at the food area divided by total number of workers in the colony. Statistical differences of the number of workers at the food area and those of worker proportion were determined by linear mixed model and Kruskal–Wallis rank sum test, respectively. In linear mixed model, removal events and colonies were treated as a fixed and random effect, respectively and the removal events was a factor in Kruskal–Wallis rank sum test.

Following the repeated removal events, the number of workers at the food area continued to decline (Kruskal–Wallis rank-sum test; χ^2^ = 8.581, *P* = 0.035). Despite reducing the number of termites at the food area, the proportion of workers at the food area remained constant throughout the experiment (LMM, Tukey’s HSD, *F*_1,12_ = 0.0223, *P* = 0.88). In the end, 20.49 ± 2.16% of all workers in the colony were found at the food area throughout all removal events (Table 1).

**Figure 1:**
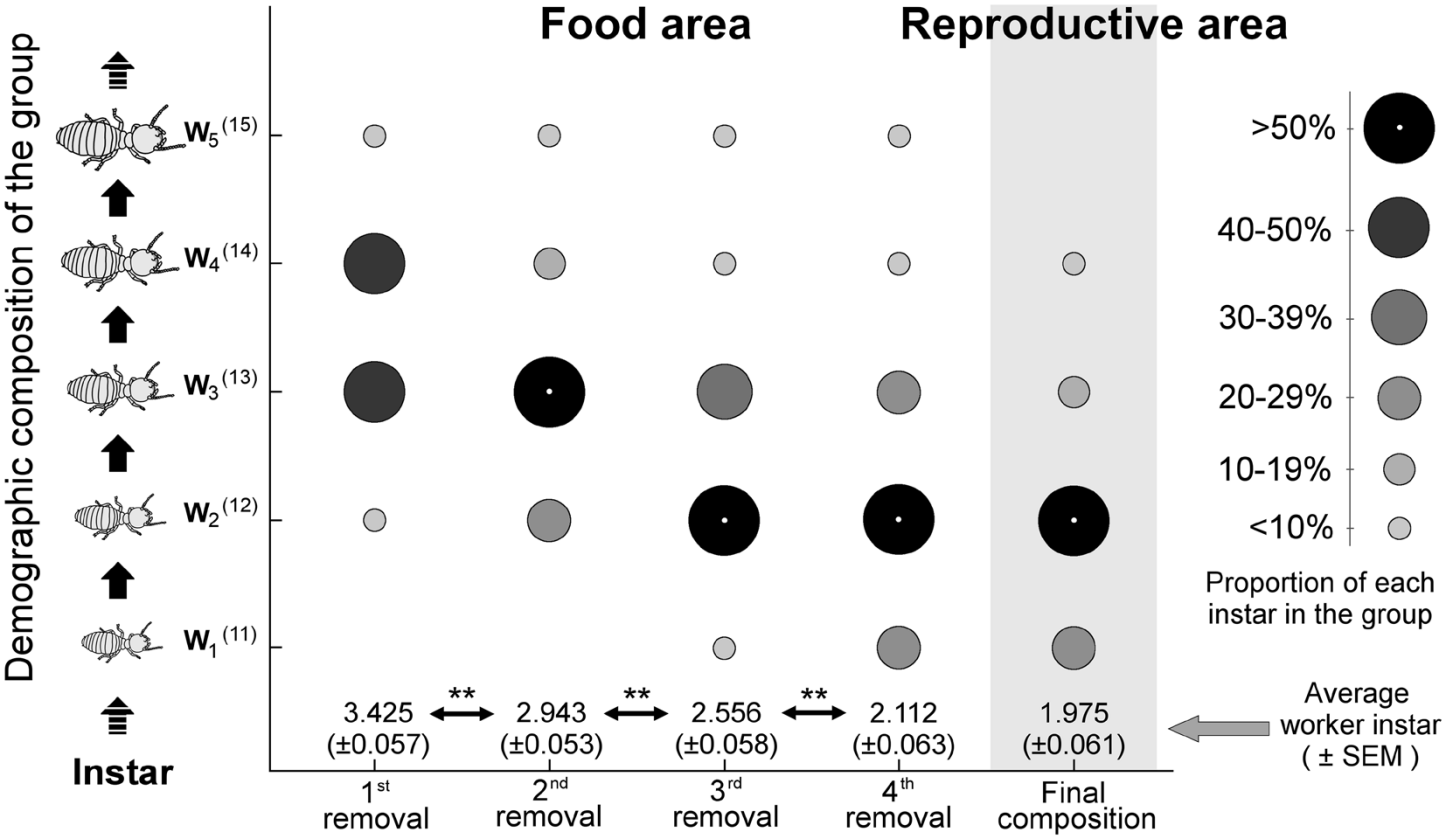
Composition of worker instars (≈ age) (circles) at the food (left) and at the reproductive area (right) after weekly removal events in *Coptotermes formosanus* colonies. Instar composition was investigated in randomly selected 40 workers for four different colonies (results were pooled) in each removal event and at the end of experiment for the composition of instars at the reproductive area. Worker instars were determined by counting the number of antennal articles followed by Chouvenc and Su (2014). The proportion of instars in each removal was determined by number of individuals in each instar divided by number of workers (*n* = 160) and the size of the circles varied depending on the percentage of worker instars. Double asterisks denote significant differences in Chi-square test (α = 0.01).

In contrast, the demographic composition of workers at the food area changed during the removal events (Fig. 1). The repeated removal of workers resulted in progressive reduction of the worker instars at the food area (average instar of workers at the food area: 1st week > 2nd week > 3rd week > 4th week; GLMM, likelihood ratio test, χ^2^ = 17.679, *P* < 0.01). At the beginning of the experiment, the average instar of workers at the food area was 3.42 (i.e., antennal articles: 13.42 ± 0.06), while it decreased to 2.11 at the end of the experiment (i.e., antennal articles: 12.11 ± 0.06). Consistently, the demographic composition changed across removal events (Pearson Chi-square test; overall comparison: χ^2^ = 349.39, *P* < 0.01; pairwise comparison, between 1st and 2nd removal: χ^2^ = 36.46, *P* < 0.01; 2nd and 3rd removal χ^2^ = 29.91, *P* < 0.01; 3rd and 4th removal: χ^2^ = 32.21, *P* < 0.01; Fig. 1). At the end of the experiment, overall colony population exhibited a relatively young demographic composition, and there was no difference in worker instar composition between the reproductive and the food area (χ^2^ = 4.47, *P* = 0.487, Fig. 1). Even though demographic composition at the food area decreased with removal events, comparison of instar composition between the food and reproductive area showed that the average instar composition at the food area was statistically higher than that of the reproductive area in each removal event, except the 4th removal (Mann–Whitney 1st removal: *U* = 19,774, *P* < 0.01; 2nd removal: *U* = 19.493, *P* < 0.01; 3rd removal: *U* = 16.758, *P* < 0.01; 4th removal *U*: 11,632, *P* = 0.120; Supplementary Fig. 1).

### Reduction in food consumption rate of replaced workers

**Figure 2:**
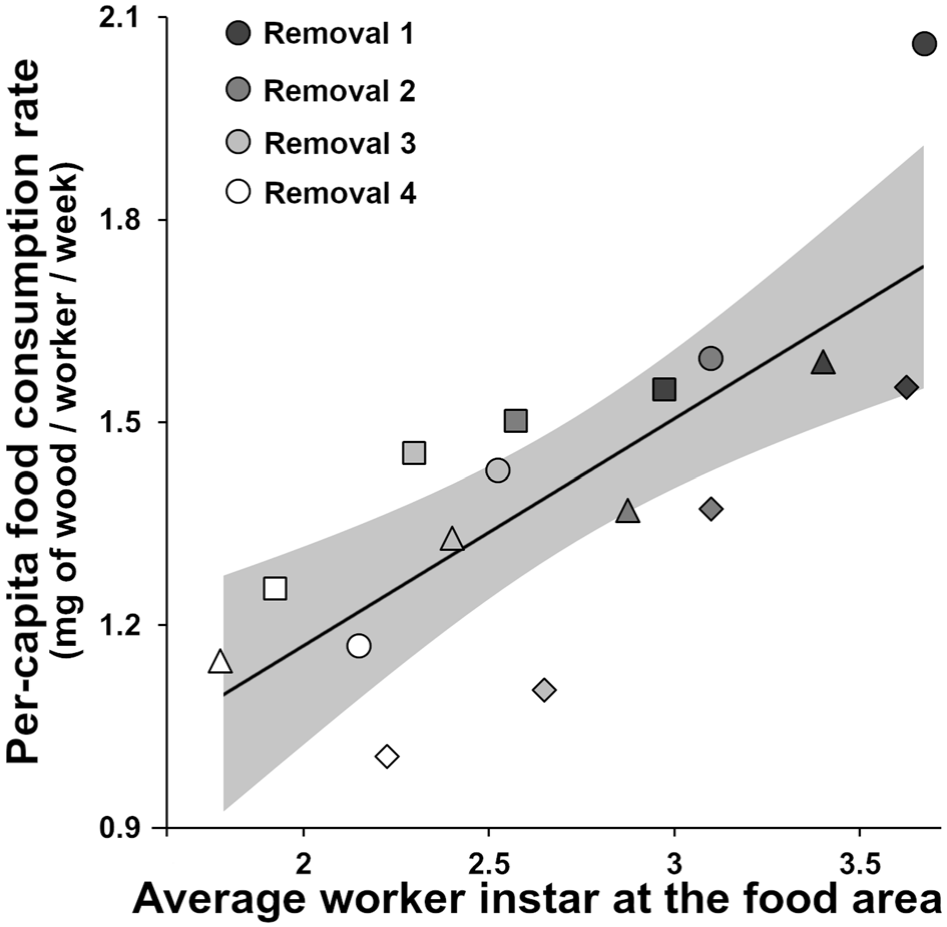
Per-capita food consumption rate (mg of wood consumption/number of workers in the colony/week) over average worker instar at the food area in *Coptotermes formosanus* colonies. The average worker instar was determined from each removal event by calculating the average from 40 individuals per colony. Square, circle, triangle and diamond shapes represent colony 1, 2, 3 and 4 respectively. Gray area and a solid line indicate 95% confidence intervals and regression line respectively.

Although the proportion of workers at the food area was constant after the series of removal events, per-capita food consumption rate decreased significantly as younger workers progressively replaced older workers (*R*^2^ = 0.588, *F* = 19.970, *P* < 0.01, Fig. 2). Colonies with a relatively young worker demographic consumed less amount of food compared to a relatively old worker demographic for food acquisition, relative to the population size of the colony.

## Discussion

Colonies of *C*. *formosanus* have the ability to maintain task allocation against loss of colony members by re-organizing spatial distribution of workers remaining within the colony. Remarkably, colonies maintained a stable ~ 20% worker proportion at the food area despite sequential removal of all workers there (Table 1). Average instar composition of workers at the food area was larger than that at reproductive area, indicating that older workers within a colony tend to depart the reproductive center and they are most likely involved in consuming wood resources (Supplementary Fig. 1). Also, as the instar composition of workers at the food area continuously declined according to removal, relatively old workers within the colony rapidly took over food acquisition role. Note that this task allocation was not very strict since food acquisition can be performed by any instar starting from W_2_, not just by a single instar group such as W_5_. Although our report is new in termites, such replacement of lost workers by other individuals is commonplace in eusocial Hymenoptera, including ants^24,50^, honey bees^23,51^, stingless bees^16^ and wasps^52^. Combined, our results indicate remarkable convergent evolution of task allocation in eusocial insects, achieved by behavioral flexibility and organized by age polyethism despite different evolutionary history to eusociality.

The sequential removal of termites resulted in a progressive reduction of worker instars pool at the food area (Fig. 1), further leading to reduction in per-capita food consumption of the colony because young workers may not be able to consume as same as old workers do (Fig. 2). Thus, in the age polyethism of subterranean termites, the older and the larger workers predominantly engage in wood consumption. When the colony loses some of these “older and larger” workers, younger workers, but relatively older than remaining colony members (e.g., W_2_ or W_3_), increase their efforts to consume food. This may maximize the efficiency of task allocation within the remaining workforce pool. Therefore, our study reveals that the proportion of colony members at the food area is actively maintained by behavioral flexibility of young workers.

This leads to the question of why does a *C. formosanus* colony have a fixed proportion of workers who leave the reproductives and focus more on wood consumption? Although replaced workers at the food area results in decreased per-capita food consumption, the colony did not compensate this depletion by increasing the proportion of workers at the food area. This stability contrasts with findings in honey bees, where colonies increased the proportion of foraging workers to compensate for the loss of old and experienced foragers^53^. Hypothetically, subterranean termite colonies may prioritize risk management of colony fragmentation over immediate maximal per-capita food consumption capacity. Due to their multiple-piece nesting that connects several feeding sites through underground tunnels^40,41^, the entire loss of feeding sites could be a natural condition facing *C. formosanus* colonies. For example, not only natural disturbances (e.g., heavy rainfall, flooding, etc.) can destroy foraging tunnels or move food resources (e.g., fallen logs) away but also predations can result in loss of termites. Since *C. formosanus* is an economically important subterranean termite^48,49^, pesticide treatments to control termites could also result in loss of foraging population. During any catastrophic loss event, the strategy of sending a fixed proportion of workers would limit the loss of colony members to a maximum of 20% of individuals within the colony, while the colony could still maintain suitable per-capita food consumption capability within the current demographic context. This highlights the importance of task allocation under unpredictable environments^54^, resulting in resilience against disturbances^26^.

Comparisons with other eusocial Hymenoptera reveal a potential difference in the modalities that regulate task allocation processes in termites; highly stable task allocation. In social Hymenoptera, catastrophic events such as loss of all foragers, can lead to long-term colony inactivity^55–59^, death of larvae^60^, or even colony death^61,62^. However, this was not the case in *C. formosanus*. Even after three successive removals of the all termites at the food area, colonies of *C. formosanus* rapidly replaced them, often within hours, and maintained a fixed proportion over time. Although we did not examine the long-term effects of removal on colony survival and productivity (i.e., further colony growth), the colonies neither collapsed nor showed cessation of food acquisition activities even after losing almost half of the colony members. Thus, termites can be more resilient compared to social Hymenoptera, which could stem from their hemimetabolous development, as termite workers are maintained as juvenile individuals^63,64^. Starting with 3rd undifferentiated instar larvae (= W_1_), individuals can readily engage in tasks for colony function, and further expand their behavioral repertoire as they age^30^. Social Hymenoptera, on the other hand, has a holometabolous development and workers are adult that went through their larval development and completed metamorphosis, which implies that the ability of a colony to readily replace foragers with relatively young individuals may depend on colony size, or may be delayed owing to the potential latency to produce new foragers^23,65^.

Although the average worker instar at the food area progressively decreased with removal events, some old workers such as W_5_ and W_4_ were still collected at the end of the experiment, despite their continuous reduction in numbers (Fig. 1). Such observations may be due to multiple factors. First, some workers might molt during the experiment as termite workers molt every 45 days and daily molting rate of the colony is about 1 to 2%^66,67^. Thus, old workers will continuously emerge regardless of removal, which is not experimentally manipulative, so that the colony could generate old workers during the experiment. Second, some old termites moved back to the nests in preparation to molt^67,68^, implying that at any given time, some relatively old workers were not at the food area. Third, some of these older workers may have been involved in food transportation from the food to the reproductive area^69^ and therefore they were not at the food area during removal events. All such factors may have contributed to the retention a small portion of relatively old individuals throughout the experiment.

In conclusion, task allocation needs to be properly regulated to meet both the demand of the colony and external conditions^13,19–22^. In this study, we showed that subterranean termites combine age polyethism and task allocation to maintain colony function when the colony members are periodically lost. By having a fixed proportion, the colony could minimize the risk of loss, while the colony could maintain the highest food consumption capability with workers within the current colony demography after the loss.

## Materials and methods

### Termite colony preparation

Colonies of *C. formosanus* were established from alate pairs (winged primary reproductives) collected during dispersal flights (May 2016) in Broward County (Florida, USA) using a light trap. Collected alates were kept in a container with moist corrugated cardboard, which favors termite self-dealation, and were brought back to the laboratory for sex determination. One hundred rearing units were prepared using plastic vials (8 cm height × 2.5 cm diameter) containing moistened soil (Timberline topsoil, Oldcastle Lawn & Garden, Inc., Atlanta, GA) at the bottom, four pieces of wood (5 × 0.5 × 0.5 cm^3^, *Picea* sp.) on top of the soil, and 3% agar^64^. The agar solution was poured over the top of wood pieces and soil to maintain moisture over time without disturbing the colony. At eight months, successful colonies were transferred to larger vials (6.3 cm height × 4.6 cm diameter) and provisioned with soil, wood, and water. Then after a year, the vial was placed in a container (1.5 L, 17 × 12 × 7 cm^3^, Pioneer Plastics, Dixon, Kentucky, USA) containing a moistened soil layer (5 cm high) and a piece of wood (14.5 × 4 × 1 cm^3^, *Picea* sp.), to allow the colony to further develop. Finally, we obtained four 3 yr-old colonies with equivalent population size for the removal experiment. The population size was initially estimated by carton nest construction and wood consumption and later confirmed through final census, from ~ 5000 to ~ 8000 termites, colony 1: 5696; colony 2: 6,961; colony 3: 7,358; colony 4: 6,997. The temperature and relative humidity were kept at 28 ± 1 °C and 80 ± 2%, respectively during the rearing period.

### Termite removal experiment

We investigated how *C. formosanus* colonies respond to the loss of colony members by weekly removing a part of the colony. Subterranean termites inhabit across multiple pieces of wood, which are connected by underground tunnels. The adult reproductives (king and queen) can only inhabit one location in the underground tunnel network while other pieces of wood contain the remaining castes, mainly workers. We simulated this by separating the experimental arena into two parts: a “reproductive area” without food where the adults were restrained from leaving using a reproductive excluder^70^ and a “food area” containing wood as the sole colony food resource accessible to all other castes. Both areas made of a 1.5 L plastic container filled with a 5 cm layer of moistened sand, and they were connected by Tygon® tubing (1 cm × 200 cm, diameter × length). At the beginning, we transferred all colony members to the reproductive area, after adding two pieces of wood as food resources to the food area (14.5 × 4 × 1 cm^3^, *Picea* sp.). Wood pieces as food were pre-weighed after being oven-dried at 70 °C for 48 h. The wood was soaked in water for 48 h before being placed into the food area. The entire arenas were covered with a black plastic sheet to prevent light disturbance throughout the experiment.

We collected all termites at the food area every seven days, by first clipping the tube at the distal end and disconnecting the food area. A new container with two pieces of wood (food area) was reconnected to the tube, allowing termites from the reproductive area to regain access to food. All collected individuals at the food area were counted by castes and preserved in 85% ethanol. For each removal event, 40 workers per colony were randomly selected from the removed termites. Worker instars (used here as a proxy for worker relative age; workers with higher instar are considered to be older) were determined by counting the number of antennal articles (16: worker 6th instar, W_6_, 15: W_5_, 14: W_4_, 13: W_3_, 12: W_2_, 11: W_1_, 10, according to Chouvenc and Su (2014) using a stereo microscope (Olympus SZX12, Tokyo, Japan). Note that no larvae (individuals smaller than W_1_) were found at food area throughout the experiments.

After the 4th removal event, we opened the reproductive area to count all individuals remaining in the colony, including those left in the connecting tube. First, we calculated proportion of workers at the food area (Table 1). To do this, we estimated the initial population size of each colony by summing up the number of individuals at the reproductive area and the cumulative number of individuals removed from the food area through the four removal events. Then, we determined the proportion of workers at the food area for each colony by number of workers at the food area divided by the estimated total number of workers in a colony at each removal event. Second, we measured the demographic composition of workers at the food area using collected termites in each removal events (Fig. 1). Using the collected termites in each removal events, we calculated proportion of each instar by number of each instar divided by total number of worker (160 workers from four different colonies) collected in each removal. For the visualization (Fig. 1), data from four colonies was pooled and proportion of worker instar was presented with different size of circles.

We also measured the weight of wood pieces at the food area after being oven-dried at 60 °C for 48 h to calculate the wood consumption by colonies throughout the experiment. By considering the number of workers and the amount of wood consumption, we calculated a “per-capita food consumption rate” (mg of wood consumption/number of workers in the colony/week) for each removal event, to estimate the food consumption capacity of workers with different instar group as removal events resulted in reduction of average worker instars at the food area. We plotted per-capita food consumption rate over the average instar in each removal.

### Statistical analysis

To investigate the effect of removal on worker instar composition at the food area, we used a generalized linear mixed model (GLMM) with Poisson distribution and log link function. The instar of individuals at the food area was fitted as the response variable, removal event (from 1st to 4th removal) was treated as a fixed effect, and the colony (*n* = 4) was included as a random effect. Statistical significance of explanatory variables was determined with likelihood ratio tests. Instar compositions of workers at the food area were also analyzed by Chi-square test (α = 0.05) to verify the overall change of compositions and between removal events. For the Chi-square analysis, data from all four colonies were pooled (*n* = 160 in each removal event). We also compared the average instar composition between the food and reproductive area with Mann–Whitney U test (α = 0.05). We estimated the instar composition at the reproductive area by sequentially adding up collected data from each removal event to the final colony census.

We also analyzed the change in the proportion of workers at the food area, using a linear mixed model (LMM) with removal events and colony origin as a fixed and random effect, respectively, followed by Tukey’s HSD test (α < 0.05) to determine statistical significances. Changes in the number of workers throughout the experiment were compared with the Kruskal–Wallis rank-sum test with removal events as a factor. Finally, a linear regression was used to determine changes in per-capita food consumption rate (response variable) over average worker instar (explanatory variable) at the food area. For this, the average worker instar was calculated by averaging instars of 40 workers per colony in each removal event. The regression line with 95% confidence intervals were plotted together to help visualize the relationship. All statistical analyses were performed using R software version 3.3.3^71^.

## Supplementary Information

Supplementary Information.
