# Supplementary material for: Age-based spatial distribution of workers is resilient to worker loss in a subterranean termite
Source: Sci Rep. 2022 May 12;12:7837. doi: 10.1038/s41598-022-11512-1 (PMC9098853; doi:10.1038/s41598-022-11512-1)

Supplementary figure 1. Average instar composition at the food area (black) and the reproductive area (white) during each removal. The instar composition at the food area was measured from 160 workers from 4 different colonies of *C. formosanus*. Due to the invisible reproductive area, the instar composition at the reproductive area was estimated from the final census of the reproductive area after all removals by sequentially adding up the previously removed termites for the final census. For example, average instar composition at the reproductive area on the first removal was calculated by combining termites from the 2nd, 3rd, 4th, and the final census. Double asterisks denote significant differences according to Mann-Whitney U test (P < 0.01). NS indicates no significant difference.


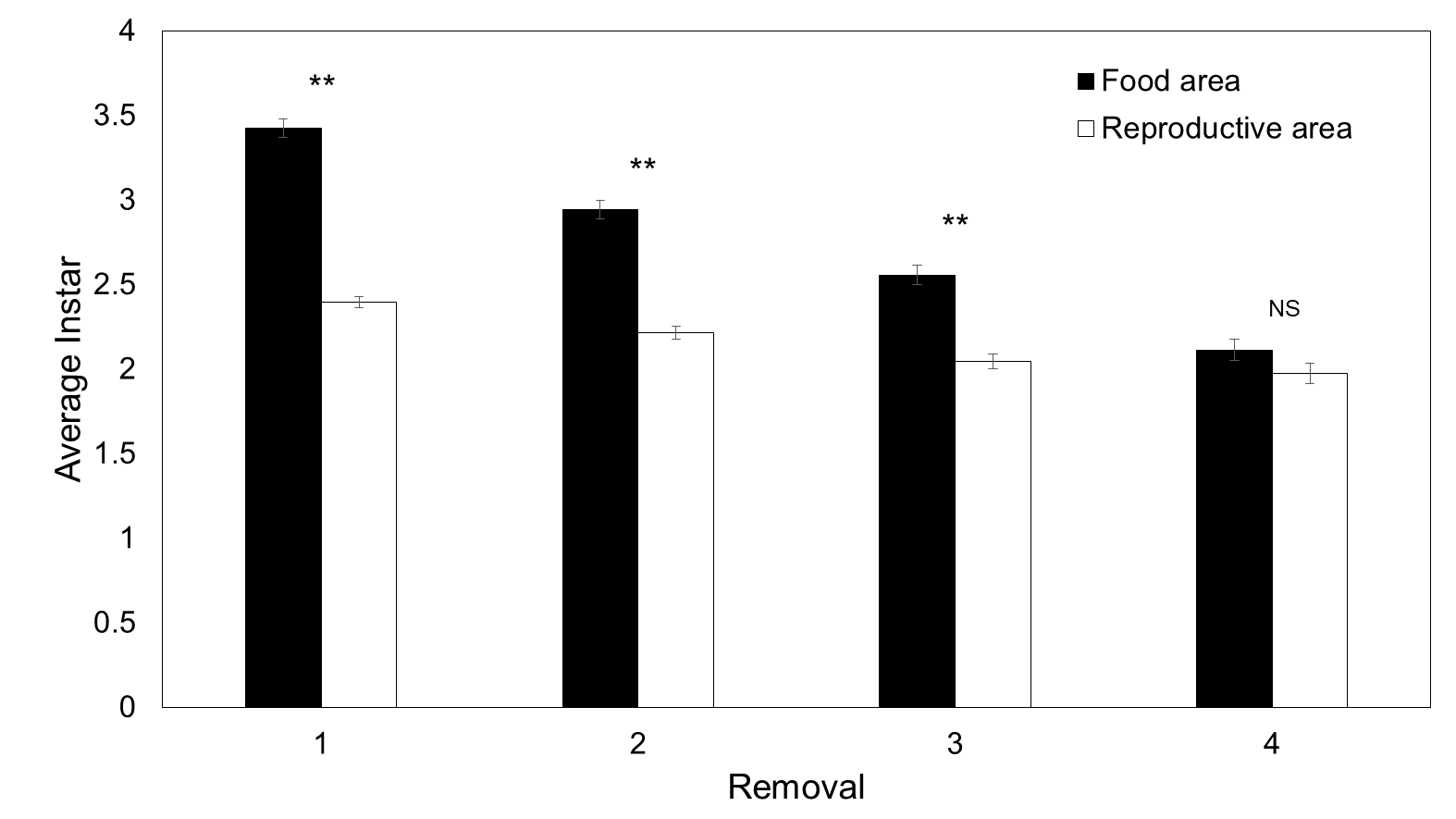

Supplement: Supplementary file 1 — Supplementary Information. [file 41598_2022_11512_MOESM1_ESM.docx]
